# Ethyl (2*Z*)-2-(3-methoxy­benzyl­idene)-7-methyl-3-oxo-5-phenyl-2,3-dihydro-5*H*-1,3-thia­zolo[3,2-*a*]pyrimidine-6-carboxyl­ate

**DOI:** 10.1107/S1600536810004812

**Published:** 2010-02-13

**Authors:** Mukesh M. Jotani, Bharat B. Baldaniya, Jerry P. Jasinski

**Affiliations:** a Department of Physics, Bhavan’s Sheth R.A. College of Science, Ahmedabad, Gujarat, 380 001, India; b Department of Chemistry, M.G. Science Institute, Navrangpura, Navrangpura, Ahmedabad, Gujarat, 380 009, India; c Department of Chemistry, Keene State College, 229 Main Street, Keene, NH 03435-2001, USA

## Abstract

In the title compound, C_24_H_22_N_2_O_4_S, the central pyrimidine ring is significantly puckered, assuming a conformation inter­mediate between a boat and a screw boat. The nearly planar thia­zole ring (r.m.s. deviation = 0.0258 Å) is fused with the pyriamidine ring, making a dihedral angle of 9.83 (7)°. The carboxyl group is in an extended conformation with an anti-periplanar orientation with respect to the dihydropyrimidine ring. The benzene ring linked at the chiral C atom is perpendicular to the pyrimidine ring [dihedral angle = 85.21 (8)°] whereas the phenyl ring is nearly coplanar, making a dihedral angle of 13.20 (8)°. An intra­molecular C—H⋯S hydrogen bond is observed. The crystal packing is influenced by weak inter­molecular C—H⋯π inter­actions and π–π stacking between the thia­zole and phenyl rings [centroid–centroid distance = 3.9656 (10) Å], which stack the mol­ecules along the *c* axis.

## Related literature

For related structures, see: Jotani & Baldaniya (2008 ▶); Sridhar *et al.* (2006 ▶); Fischer *et al.* (2007 ▶); Baldaniya & Jotani (2008 ▶); Jotani *et al.* (2009 ▶). For the biological activity of dihydro­pyrimidines, see: Wichmann *et al.* (1999 ▶); Kappe (2000 ▶); Mayer *et al.* (1999 ▶). For a description of the Cambridge Structural Database, see: Allen, (2002 ▶). For hydrogen-bond motifs, see: Bernstein *et al.* (1995 ▶). For puckering parameters, see: Cremer & Pople (1975 ▶).
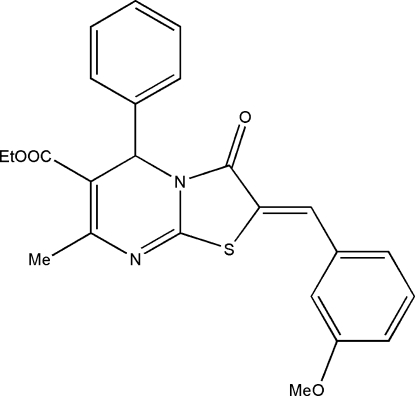


## Experimental

### 

#### Crystal data

C_24_H_22_N_2_O_4_S
*M* *_r_* = 434.50
Monoclinic, 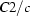

*a* = 33.0445 (6) Å
*b* = 9.5013 (2) Å
*c* = 13.8845 (2) Å
β = 101.548 (1)°
*V* = 4271.01 (13) Å^3^
*Z* = 8
Mo *K*α radiation
μ = 0.19 mm^−1^
*T* = 293 K
0.30 × 0.20 × 0.15 mm

#### Data collection

Bruker APEXII CCD diffractometer
Absorption correction: multi-scan (*SADABS*; Sheldrick, 1996 ▶) *T*_min_ = 0.946, *T*_max_ = 0.973
27172 measured reflections
6222 independent reflections
4099 reflections with *I* > 2σ(*I*)
*R*_int_ = 0.035

#### Refinement

*R*[*F* ^2^ > 2σ(*F* ^2^)] = 0.043
*wR*(*F* ^2^) = 0.113
*S* = 0.93
6222 reflections
284 parameters
H-atom parameters constrained
Δρ_max_ = 0.24 e Å^−3^
Δρ_min_ = −0.23 e Å^−3^

### 

Data collection: *APEX2* (Bruker, 2004 ▶); cell refinement: *APEX2* and *SAINT* (Bruker, 2004 ▶); data reduction: *SAINT* and *XPREP* (Bruker, 2004 ▶); program(s) used to solve structure: *SIR97* (Altomare *et al.*, 1999 ▶); program(s) used to refine structure: *SHELXL97* (Sheldrick, 2008 ▶); molecular graphics: *PLATON* (Spek, 2009 ▶); software used to prepare material for publication: *PLATON*.

## Supplementary Material

Crystal structure: contains datablocks global, I. DOI: 10.1107/S1600536810004812/ng2731sup1.cif

Structure factors: contains datablocks I. DOI: 10.1107/S1600536810004812/ng2731Isup2.hkl

Additional supplementary materials:  crystallographic information; 3D view; checkCIF report

## Figures and Tables

**Table 1:** Hydrogen-bond geometry (Å, °) *Cg*3 is the centroid of the C11–C16 ring.

*D*—H⋯*A*	*D*—H	H⋯*A*	*D*⋯*A*	*D*—H⋯*A*
C19—H19⋯S1	0.93	2.54	3.2561 (15)	134
C10—H10*B*⋯*Cg*3^i^	0.96	2.87	3.755 (3)	153
C21—H21⋯*Cg*3^ii^	0.93	2.79	3.602 (2)	147

Symmetry codes: (i) 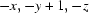; (ii) 
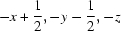
.

**Fig. 1.**
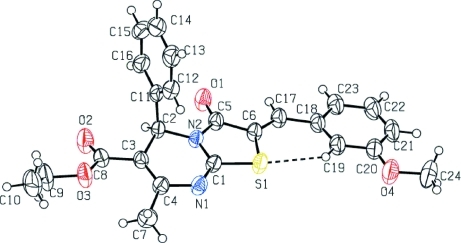
The molecular structure of (I), showing 50% probability displacement ellipsoids. Dashed line indicates an intramolecular bond.

**Fig. 2.**
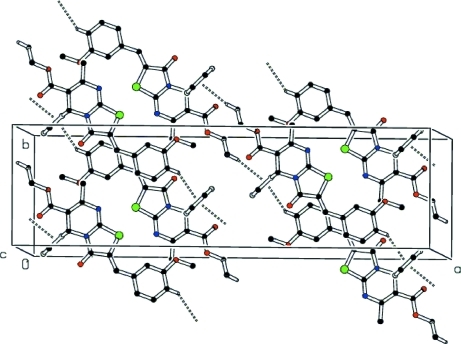
PLATON (Spek, 2009) plot of (I), showing weak C—H···π intermolecular interactions as dashed lines. H atoms not involved in hydrogen bonding have been omitted.

**Fig. 3.**
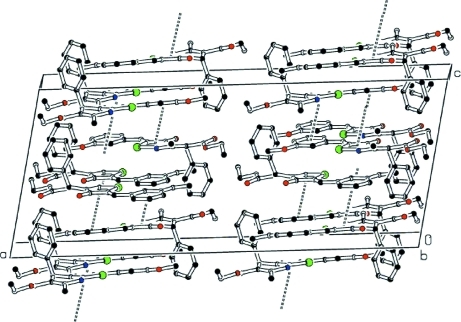
A view of the π-π stacking interaction (dashed line) in the crystal structure of (I). H atoms have been omitted for clarity.

## supplementary crystallographic information

### Comment

Series of fused thiazolo [3,2-a] pyrimidine derivatives are antagonistic to group 2 mGlu receptors depending upon substitution of two phenyl rings at the 2 and 5 positions as well as substituents at positions 6 and 7 of the scaffold (Wichmann *et al.*, 1999). Moreover dihydropyrimidines (DHPMs) have  remarkable potency with antiviral, antitumor, antibacterial and *anti*-inflammatory activities, and are used as antihypertensive agents and calcium channel modulators (Kappe, 2000). A DHPM analog has been identified as a potential anticancer lead that is involved in blocking mitosis by inhibition of a kinesin motor protein (Mayer *et al.*, 1999).  In continuation of our studies on a series of pharmacologically interesting thiazolo [3,2-a] pyrimidine derivatives (Jotani & Baldaniya, 2008; Baldaniya & Jotani, 2008; Jotani *et al.*, 2009) to examine the effect of substituents of varying size from the phenyl ring on crystal packing, a crystal structure of the title compound, C_24_H_22_N_2_O_4_S, (I), is reported.

In (I), the central pyrimidine ring with a chiral C2 atom at the point of substitution of a benzene ring (C11-C16) is significantly puckered and adopts a conformation which is best described as an intermediate between a boat and screw boat form, Fig. 1. The ring puckering parameters (Cremer & Pople, 1975) for the pyrimidine ring are q2 = 0.1997 (14) Å, q3 = 0.0575 (17)Å, Q = 0.2090 (15) Å; θ = 74.1 (4) ° and φ = 157.9 (4)°.  Idealized values for a boat and screw boat conformation are: θ = 90° and 67.5°, and φ = 60k and (60k + 30)°, respectively, where k is an integer.  The fusion of a nearly planar thiazole ring (r.m.s. deviation = 0.0258Å) results in slightly deviated N1—C1 and N1—C4 bond lengths in the pyrimidine ring.  The fused thiazole ring has geometrical parameters similar to analagous structures (Jotani & Baldaniya, 2008; Baldaniya & Jotani, 2008; Jotani *et al.*, 2009; Sridhar *et al.*, 2006; Fischer *et al.*, 2007).   The dihedral angles between the mean planes of the benzene (C11-C16) and 3-methoxy phenyl ring (C18-C23) substituted at the carbon C6 atom and the pyrimidine ring are 85.21 (8)° and 13.20 (8)° respectively. The dihedral angle between the mean planes of the benzene (C11-C16) and 3-methoxy phenyl rings (C18-C23) is 87.73°.  The carboxyl group linked at C3 atom is in an extended conformation with an anti-periplanar orientation with respect to pyrimidine ring.  The ethyl group remains nearly planar (C8/O3/C9/C10 = -156.87 (19)° resulting a trans conformation of ethoxy group with respect to O2—C9 bond. A short intramolecular C—H···S hydrogen bond between the phenyl carbon C19 and thiazolo sulphur S1 (Fig. 2 & Table 1) forms a pseudo-six-membered ring of S(6) graph-set motif (Bernstein *et al.*,  1995) which helps to consolidate the crystal packing.  C-H···Cg π interactions exist between a carboxylate carbon atom and benzene (Cg3) [C10···H10···Cg3], and between a phenyl carbon atom and benzene (Cg3) [C21—H21···Cg3], (Table 2). In addition a weak π–π intermolecular interaction is observed between the ring centroids of the thiazole (Cg1) and phenyl (Cg4) rings (Fig. 3, Table 3).

### Experimental

A mixture of ethyl 6-methyl-4-phenyl-2-thioxo-1,2,3,4-tetrahydropyrimidine-5-carboxylate (0.01 mol), chloroacetic acid (0.01 mol), fused sodium acetate (6 g m) in glacial acetic acid (25 ml), acetic anhydride (10 ml) and 3-methoxy benzaldehyde (0.01 mol) was refluxed for 3 hours. The reaction mixture was cooled and poured into cold water. The resulting solid was collected and crystallized from methanol to obtain the final product (84 % yield, mp 423 K). The compound was recrystallized by slow evaporation of a benzene-ethanol (8:2) solution, yielding colorless, single crystals suitable for X-ray diffraction.

### Refinement

H atoms were placed in idealized positions (C—H = 0.93— 0.98 Å) and constrained to ride on their parent atoms with *U*_iso_(H) = 1.5*U*_eq_(C) for methyl H atoms and 1.2*U*_eq_(C) for other H atoms.

### Crystal data

**Table:**

C_24_H_22_N_2_O_4_S	*F*(000) = 1824
*M**_r_* = 434.50	*D*_x_ = 1.351 Mg m^−^^3^
Monoclinic, *C*2/*c*	Mo *K*α radiation, λ = 0.71069 Å
Hall symbol: -C 2yc	Cell parameters from 5670 reflections
*a* = 33.0445 (6) Å	θ = 3.0–30.0°
*b* = 9.5013 (2) Å	µ = 0.19 mm^−^^1^
*c* = 13.8845 (2) Å	*T* = 293 K
β = 101.548 (1)°	Plate, colorless
*V* = 4271.01 (13) Å^3^	0.30 × 0.20 × 0.15 mm
*Z* = 8	

### Data collection

**Table:**

Bruker APEXII CCD diffractometer	6222 independent reflections
Radiation source: fine-focus sealed tube	4099 reflections with *I* > 2σ(*I*)
graphite	*R*_int_ = 0.035
ω and φ scans	θ_max_ = 30.0°, θ_min_ = 2.2°
Absorption correction: multi-scan (*SADABS*; Sheldrick, 1996)	*h* = −46→46
*T*_min_ = 0.946, *T*_max_ = 0.973	*k* = −13→13
27172 measured reflections	*l* = −19→19

### Refinement

**Table:**

Refinement on *F*^2^	Secondary atom site location: difference Fourier map
Least-squares matrix: full	Hydrogen site location: inferred from neighbouring sites
*R*[*F*^2^ > 2σ(*F*^2^)] = 0.043	H-atom parameters constrained
*wR*(*F*^2^) = 0.113	*w* = 1/[σ^2^(*F*_o_^2^) + (0.049*P*)^2^ + 2.3025*P*] where *P* = (*F*_o_^2^ + 2*F*_c_^2^)/3
*S* = 0.93	(Δ/σ)_max_ < 0.001
6222 reflections	Δρ_max_ = 0.24 e Å^−^^3^
284 parameters	Δρ_min_ = −0.23 e Å^−^^3^
0 restraints	Extinction correction: *SHELXL*, Fc^*^=kFc[1+0.001xFc^2^λ^3^/sin(2θ)]^-1/4^
Primary atom site location: structure-invariant direct methods	Extinction coefficient: 0.00104 (16)

### Special details

**Table:**

Geometry. All esds (except the esd in the dihedral angle between two l.s. planes) are estimated using the full covariance matrix. The cell esds are taken into account individually in the estimation of esds in distances, angles and torsion angles; correlations between esds in cell parameters are only used when they are defined by crystal symmetry. An approximate (isotropic) treatment of cell esds is used for estimating esds involving l.s. planes.
Refinement. Refinement of *F*^2^ against ALL reflections. The weighted *R*-factor *wR* and goodness of fit *S* are based on *F*^2^, conventional *R*-factors *R* are based on *F*, with *F* set to zero for negative *F*^2^. The threshold expression of *F*^2^ > σ(*F*^2^) is used only for calculating *R*-factors(gt) *etc*. and is not relevant to the choice of reflections for refinement. *R*-factors based on *F*^2^ are statistically about twice as large as those based on *F*, and *R*- factors based on ALL data will be even larger.

### Fractional atomic coordinates and isotropic or equivalent isotropic displacement parameters (Å^2^)

**Table:**

	*x*	*y*	*z*	*U*_iso_*/*U*_eq_	
S1	0.235672 (11)	0.12268 (4)	0.10790 (3)	0.05063 (12)	
N1	0.18618 (4)	0.34110 (13)	0.11604 (10)	0.0487 (3)	
N2	0.15837 (3)	0.11297 (12)	0.11518 (9)	0.0373 (2)	
O1	0.14512 (3)	−0.11959 (11)	0.12997 (10)	0.0571 (3)	
O2	0.04874 (4)	0.27636 (13)	0.15987 (10)	0.0672 (4)	
O3	0.06592 (4)	0.49424 (12)	0.12836 (10)	0.0629 (3)	
O4	0.37128 (4)	−0.07609 (15)	0.09922 (11)	0.0733 (4)	
C1	0.18927 (4)	0.20741 (15)	0.11242 (11)	0.0406 (3)	
C2	0.11507 (4)	0.15838 (14)	0.10056 (10)	0.0368 (3)	
H2	0.1018	0.1070	0.1470	0.044*	
C3	0.11429 (4)	0.31329 (14)	0.12389 (10)	0.0381 (3)	
C4	0.14806 (4)	0.39460 (15)	0.13134 (11)	0.0432 (3)	
C5	0.16927 (4)	−0.02682 (15)	0.12298 (11)	0.0411 (3)	
C6	0.21375 (4)	−0.04258 (15)	0.12057 (11)	0.0416 (3)	
C7	0.15201 (5)	0.54599 (17)	0.16009 (15)	0.0617 (5)	
H7A	0.1494	0.6032	0.1022	0.093*	
H7B	0.1785	0.5619	0.2018	0.093*	
H7C	0.1306	0.5704	0.1948	0.093*	
C8	0.07349 (5)	0.35749 (15)	0.14060 (11)	0.0426 (3)	
C9	0.02824 (6)	0.5474 (2)	0.15207 (18)	0.0759 (6)	
H9A	0.0321	0.5645	0.2222	0.091*	
H9B	0.0063	0.4787	0.1340	0.091*	
C10	0.01708 (7)	0.6780 (2)	0.09834 (18)	0.0874 (7)	
H10A	0.0398	0.7426	0.1124	0.131*	
H10B	−0.0066	0.7186	0.1182	0.131*	
H10C	0.0106	0.6587	0.0291	0.131*	
C11	0.09283 (4)	0.12247 (14)	−0.00271 (11)	0.0394 (3)	
C12	0.10440 (5)	0.18604 (18)	−0.08289 (12)	0.0504 (4)	
H12	0.1246	0.2555	−0.0730	0.060*	
C13	0.08613 (5)	0.1470 (2)	−0.17714 (13)	0.0638 (5)	
H13	0.0945	0.1883	−0.2306	0.077*	
C14	0.05548 (6)	0.0471 (2)	−0.19211 (15)	0.0670 (5)	
H14	0.0431	0.0210	−0.2558	0.080*	
C15	0.04305 (5)	−0.0141 (2)	−0.11405 (16)	0.0651 (5)	
H15	0.0221	−0.0809	−0.1246	0.078*	
C16	0.06171 (5)	0.02327 (16)	−0.01906 (13)	0.0509 (4)	
H16	0.0532	−0.0188	0.0340	0.061*	
C17	0.23076 (4)	−0.16995 (17)	0.12650 (11)	0.0459 (3)	
H17	0.2127	−0.2427	0.1335	0.055*	
C18	0.27255 (4)	−0.21580 (16)	0.12404 (11)	0.0452 (3)	
C19	0.30375 (5)	−0.12586 (17)	0.11084 (12)	0.0506 (4)	
H19	0.2983	−0.0302	0.1020	0.061*	
C20	0.34317 (5)	−0.1759 (2)	0.11051 (12)	0.0533 (4)	
C21	0.35128 (5)	−0.3184 (2)	0.12200 (12)	0.0588 (4)	
H21	0.3776	−0.3529	0.1219	0.071*	
C22	0.32012 (5)	−0.4086 (2)	0.13349 (13)	0.0597 (4)	
H22	0.3255	−0.5045	0.1406	0.072*	
C23	0.28096 (5)	−0.35955 (17)	0.13471 (12)	0.0520 (4)	
H23	0.2602	−0.4220	0.1426	0.062*	
C24	0.41086 (5)	−0.1219 (3)	0.08662 (16)	0.0806 (6)	
H24A	0.4245	−0.1705	0.1447	0.121*	
H24B	0.4270	−0.0419	0.0755	0.121*	
H24C	0.4077	−0.1843	0.0312	0.121*	

### Atomic displacement parameters (Å^2^)

**Table:**

	*U*^11^	*U*^22^	*U*^33^	*U*^12^	*U*^13^	*U*^23^
S1	0.03193 (18)	0.0440 (2)	0.0776 (3)	0.00373 (15)	0.01474 (17)	0.00509 (18)
N1	0.0357 (6)	0.0366 (7)	0.0741 (9)	−0.0006 (5)	0.0117 (6)	0.0037 (6)
N2	0.0287 (5)	0.0328 (6)	0.0502 (7)	0.0034 (4)	0.0072 (4)	0.0033 (5)
O1	0.0435 (6)	0.0346 (6)	0.0973 (9)	0.0032 (5)	0.0237 (6)	0.0075 (5)
O2	0.0542 (7)	0.0474 (7)	0.1104 (10)	0.0035 (6)	0.0416 (7)	0.0055 (6)
O3	0.0529 (7)	0.0417 (6)	0.1035 (10)	0.0146 (5)	0.0386 (6)	0.0074 (6)
O4	0.0429 (6)	0.0804 (9)	0.1015 (10)	0.0056 (6)	0.0259 (6)	0.0009 (8)
C1	0.0314 (6)	0.0387 (8)	0.0508 (8)	0.0015 (6)	0.0063 (6)	0.0034 (6)
C2	0.0287 (6)	0.0331 (7)	0.0503 (8)	0.0036 (5)	0.0120 (5)	0.0034 (6)
C3	0.0365 (7)	0.0325 (7)	0.0464 (8)	0.0055 (5)	0.0109 (6)	0.0019 (6)
C4	0.0397 (7)	0.0324 (7)	0.0571 (9)	0.0033 (6)	0.0087 (6)	0.0029 (6)
C5	0.0360 (7)	0.0346 (7)	0.0533 (8)	0.0060 (6)	0.0104 (6)	0.0037 (6)
C6	0.0337 (7)	0.0416 (8)	0.0501 (8)	0.0059 (6)	0.0093 (6)	0.0036 (6)
C7	0.0529 (10)	0.0340 (8)	0.0951 (14)	−0.0007 (7)	0.0073 (9)	−0.0062 (8)
C8	0.0427 (7)	0.0383 (8)	0.0497 (8)	0.0064 (6)	0.0168 (6)	0.0002 (6)
C9	0.0631 (11)	0.0580 (12)	0.1196 (17)	0.0211 (9)	0.0493 (12)	0.0010 (11)
C10	0.0741 (14)	0.0789 (15)	0.1087 (17)	0.0371 (12)	0.0170 (12)	−0.0026 (13)
C11	0.0289 (6)	0.0341 (7)	0.0551 (8)	0.0075 (5)	0.0081 (6)	−0.0022 (6)
C12	0.0399 (8)	0.0559 (10)	0.0548 (9)	0.0037 (7)	0.0080 (7)	−0.0002 (7)
C13	0.0489 (9)	0.0858 (14)	0.0555 (10)	0.0150 (9)	0.0071 (8)	−0.0010 (9)
C14	0.0511 (10)	0.0773 (13)	0.0652 (12)	0.0198 (9)	−0.0063 (8)	−0.0185 (10)
C15	0.0399 (8)	0.0525 (10)	0.0941 (15)	0.0035 (8)	−0.0075 (9)	−0.0161 (10)
C16	0.0366 (7)	0.0409 (8)	0.0733 (11)	0.0023 (6)	0.0062 (7)	−0.0022 (7)
C17	0.0378 (7)	0.0429 (8)	0.0576 (9)	0.0083 (6)	0.0111 (6)	0.0028 (7)
C18	0.0397 (7)	0.0486 (9)	0.0469 (8)	0.0140 (6)	0.0078 (6)	0.0005 (6)
C19	0.0414 (8)	0.0504 (9)	0.0605 (10)	0.0125 (7)	0.0116 (7)	0.0005 (7)
C20	0.0401 (8)	0.0688 (11)	0.0517 (9)	0.0100 (8)	0.0109 (7)	−0.0019 (8)
C21	0.0454 (9)	0.0744 (12)	0.0569 (10)	0.0266 (9)	0.0105 (7)	−0.0012 (8)
C22	0.0580 (10)	0.0561 (10)	0.0651 (11)	0.0262 (8)	0.0124 (8)	0.0025 (8)
C23	0.0487 (9)	0.0512 (9)	0.0568 (9)	0.0146 (7)	0.0122 (7)	0.0012 (7)
C24	0.0394 (9)	0.1200 (19)	0.0849 (14)	0.0135 (11)	0.0187 (9)	0.0131 (12)

### Geometric parameters (Å, °)

**Table:**

S1—C1	1.7439 (14)	C10—H10B	0.9600
S1—C6	1.7528 (15)	C10—H10C	0.9600
N1—C1	1.2761 (19)	C11—C16	1.380 (2)
N1—C4	1.4134 (18)	C11—C12	1.386 (2)
N2—C1	1.3655 (17)	C12—C13	1.378 (2)
N2—C5	1.3747 (17)	C12—H12	0.9300
N2—C2	1.4693 (16)	C13—C14	1.374 (3)
O1—C5	1.2059 (17)	C13—H13	0.9300
O2—C8	1.1928 (18)	C14—C15	1.364 (3)
O3—C8	1.3277 (17)	C14—H14	0.9300
O3—C9	1.4413 (18)	C15—C16	1.386 (2)
O4—C20	1.358 (2)	C15—H15	0.9300
O4—C24	1.422 (2)	C16—H16	0.9300
C2—C3	1.5084 (19)	C17—C18	1.4551 (19)
C2—C11	1.513 (2)	C17—H17	0.9300
C2—H2	0.9800	C18—C19	1.379 (2)
C3—C4	1.3441 (19)	C18—C23	1.396 (2)
C3—C8	1.4740 (19)	C19—C20	1.388 (2)
C4—C7	1.491 (2)	C19—H19	0.9300
C5—C6	1.4842 (19)	C20—C21	1.383 (3)
C6—C17	1.330 (2)	C21—C22	1.373 (3)
C7—H7A	0.9600	C21—H21	0.9300
C7—H7B	0.9600	C22—C23	1.379 (2)
C7—H7C	0.9600	C22—H22	0.9300
C9—C10	1.457 (3)	C23—H23	0.9300
C9—H9A	0.9700	C24—H24A	0.9600
C9—H9B	0.9700	C24—H24B	0.9600
C10—H10A	0.9600	C24—H24C	0.9600
			
C1—S1—C6	91.48 (7)	H10A—C10—H10C	109.5
C1—N1—C4	116.41 (12)	H10B—C10—H10C	109.5
C1—N2—C5	116.84 (11)	C16—C11—C12	118.81 (14)
C1—N2—C2	121.08 (11)	C16—C11—C2	120.96 (13)
C5—N2—C2	121.81 (11)	C12—C11—C2	120.20 (13)
C8—O3—C9	117.58 (13)	C13—C12—C11	120.45 (16)
C20—O4—C24	117.87 (16)	C13—C12—H12	119.8
N1—C1—N2	125.93 (13)	C11—C12—H12	119.8
N1—C1—S1	122.60 (11)	C14—C13—C12	119.96 (18)
N2—C1—S1	111.43 (10)	C14—C13—H13	120.0
N2—C2—C3	108.35 (11)	C12—C13—H13	120.0
N2—C2—C11	109.85 (10)	C15—C14—C13	120.32 (17)
C3—C2—C11	113.42 (11)	C15—C14—H14	119.8
N2—C2—H2	108.4	C13—C14—H14	119.8
C3—C2—H2	108.4	C14—C15—C16	119.98 (17)
C11—C2—H2	108.4	C14—C15—H15	120.0
C4—C3—C8	126.55 (13)	C16—C15—H15	120.0
C4—C3—C2	121.85 (12)	C11—C16—C15	120.45 (16)
C8—C3—C2	111.55 (12)	C11—C16—H16	119.8
C3—C4—N1	122.20 (13)	C15—C16—H16	119.8
C3—C4—C7	127.00 (14)	C6—C17—C18	131.48 (15)
N1—C4—C7	110.74 (13)	C6—C17—H17	114.3
O1—C5—N2	123.05 (12)	C18—C17—H17	114.3
O1—C5—C6	127.03 (13)	C19—C18—C23	118.83 (14)
N2—C5—C6	109.91 (12)	C19—C18—C17	123.77 (14)
C17—C6—C5	119.90 (13)	C23—C18—C17	117.40 (15)
C17—C6—S1	130.03 (11)	C18—C19—C20	121.03 (15)
C5—C6—S1	110.06 (10)	C18—C19—H19	119.5
C4—C7—H7A	109.5	C20—C19—H19	119.5
C4—C7—H7B	109.5	O4—C20—C21	125.14 (15)
H7A—C7—H7B	109.5	O4—C20—C19	115.18 (16)
C4—C7—H7C	109.5	C21—C20—C19	119.68 (16)
H7A—C7—H7C	109.5	C22—C21—C20	119.45 (15)
H7B—C7—H7C	109.5	C22—C21—H21	120.3
O2—C8—O3	122.74 (13)	C20—C21—H21	120.3
O2—C8—C3	122.77 (13)	C21—C22—C23	121.21 (16)
O3—C8—C3	114.43 (13)	C21—C22—H22	119.4
O3—C9—C10	108.86 (16)	C23—C22—H22	119.4
O3—C9—H9A	109.9	C22—C23—C18	119.79 (17)
C10—C9—H9A	109.9	C22—C23—H23	120.1
O3—C9—H9B	109.9	C18—C23—H23	120.1
C10—C9—H9B	109.9	O4—C24—H24A	109.5
H9A—C9—H9B	108.3	O4—C24—H24B	109.5
C9—C10—H10A	109.5	H24A—C24—H24B	109.5
C9—C10—H10B	109.5	O4—C24—H24C	109.5
H10A—C10—H10B	109.5	H24A—C24—H24C	109.5
C9—C10—H10C	109.5	H24B—C24—H24C	109.5
			
C4—N1—C1—N2	4.8 (2)	C4—C3—C8—O2	−158.39 (16)
C4—N1—C1—S1	−172.62 (11)	C2—C3—C8—O2	19.1 (2)
C5—N2—C1—N1	−171.96 (14)	C4—C3—C8—O3	24.3 (2)
C2—N2—C1—N1	14.0 (2)	C2—C3—C8—O3	−158.23 (13)
C5—N2—C1—S1	5.71 (16)	C8—O3—C9—C10	−156.88 (18)
C2—N2—C1—S1	−168.38 (10)	N2—C2—C11—C16	113.42 (13)
C6—S1—C1—N1	172.96 (14)	C3—C2—C11—C16	−125.17 (14)
C6—S1—C1—N2	−4.80 (11)	N2—C2—C11—C12	−64.54 (16)
C1—N2—C2—C3	−22.21 (17)	C3—C2—C11—C12	56.87 (16)
C5—N2—C2—C3	164.00 (12)	C16—C11—C12—C13	−2.3 (2)
C1—N2—C2—C11	102.20 (14)	C2—C11—C12—C13	175.70 (14)
C5—N2—C2—C11	−71.60 (16)	C11—C12—C13—C14	1.7 (3)
N2—C2—C3—C4	15.01 (18)	C12—C13—C14—C15	−0.1 (3)
C11—C2—C3—C4	−107.23 (15)	C13—C14—C15—C16	−0.8 (3)
N2—C2—C3—C8	−162.59 (11)	C12—C11—C16—C15	1.4 (2)
C11—C2—C3—C8	75.16 (15)	C2—C11—C16—C15	−176.60 (13)
C8—C3—C4—N1	178.44 (14)	C14—C15—C16—C11	0.1 (2)
C2—C3—C4—N1	1.2 (2)	C5—C6—C17—C18	178.62 (15)
C8—C3—C4—C7	1.4 (3)	S1—C6—C17—C18	−0.4 (3)
C2—C3—C4—C7	−175.82 (15)	C6—C17—C18—C19	−2.3 (3)
C1—N1—C4—C3	−12.3 (2)	C6—C17—C18—C23	178.40 (16)
C1—N1—C4—C7	165.13 (14)	C23—C18—C19—C20	−1.6 (2)
C1—N2—C5—O1	177.02 (14)	C17—C18—C19—C20	179.12 (15)
C2—N2—C5—O1	−8.9 (2)	C24—O4—C20—C21	7.8 (3)
C1—N2—C5—C6	−3.43 (18)	C24—O4—C20—C19	−172.72 (16)
C2—N2—C5—C6	170.62 (12)	C18—C19—C20—O4	−178.46 (15)
O1—C5—C6—C17	0.0 (2)	C18—C19—C20—C21	1.1 (2)
N2—C5—C6—C17	−179.57 (13)	O4—C20—C21—C22	179.52 (16)
O1—C5—C6—S1	179.16 (14)	C19—C20—C21—C22	0.1 (2)
N2—C5—C6—S1	−0.38 (15)	C20—C21—C22—C23	−0.6 (3)
C1—S1—C6—C17	−178.02 (15)	C21—C22—C23—C18	0.0 (3)
C1—S1—C6—C5	2.89 (11)	C19—C18—C23—C22	1.0 (2)
C9—O3—C8—O2	8.2 (3)	C17—C18—C23—C22	−179.63 (15)
C9—O3—C8—C3	−174.50 (16)		

### Hydrogen-bond geometry (Å, °)

**Table:**

Cg3 is the centroid of the C11–C16 ring.

**Table:**

*D*—H···*A*	*D*—H	H···*A*	*D*···*A*	*D*—H···*A*
C19—H19···S1	0.93	2.54	3.2561 (15)	134
C10—H10B···Cg3^i^	0.96	2.87	3.755 (3)	153
C21—H21···Cg3^ii^	0.93	2.79	3.602 (2)	147

Symmetry codes: (i) −*x*, −*y*+1, −*z*; (ii) −*x*+1/2, −*y*−1/2, −*z*.

### Table 2 π-π Hydrogen-bond geometry (Å, °) for (I).

**Table:**

Cg1···Cg4^iii^	3.9656 (10)

Symmetry codes: (iii) 1/2-x,1/2+y,1/2-z; Cg1 and Cg4 are the centroids of the S1/C1/N2/C5/C6 amd C18—C23 rings.
